# Visualizing the fibre texture of satin spar using laboratory 2D X-ray diffraction

**DOI:** 10.1107/S1600576724000529

**Published:** 2024-02-12

**Authors:** Xiaodong (Tony) Wang, Christoph Schrank, Michael Jones

**Affiliations:** a Central Analytical Research Facility, Queensland University of Technology, Brisbane, Queensland 4001, Australia; b Centre for Materials Science, Queensland University of Technology, Brisbane, Queensland 4001, Australia; c School of Earth and Atmospheric Sciences, Queensland University of Technology, Brisbane, Queensland 4001, Australia; d Centre for Data Science, Queensland University of Technology, Brisbane, Queensland 4001, Australia; e Planetary Surface Exploration Group, Queensland University of Technology, Brisbane, Queensland 4001, Australia; f School of Chemistry and Physics, Queensland University of Technology, Brisbane, Queensland 4001, Australia; Montanuniversität Leoben, Austria

**Keywords:** fibre texture, satin spar, wide-range reciprocal space mapping, WR-RSM, 2D X-ray diffraction, gypsum

## Abstract

The suitability of point focus X-ray beam and area detector techniques for the determination of the uniaxial symmetry axis (fibre texture) of the natural mineral satin spar is demonstrated. Among the various diffraction techniques used in this report, including powder diffraction, 2D pole figures, rocking curves looped on φ and 2D X-ray diffraction, a single simple symmetric 2D scan collecting the reciprocal plane perpendicular to the apparent fibre axis provided sufficient information to determine the crystallographic orientation of the fibre axis. A geometrical explanation of the ‘wing’ feature formed by diffraction spots from the fibre-textured satin spar in 2D scans is provided. The technique of wide-range reciprocal space mapping restores the ‘wing’ featured diffraction spots on the 2D detector back to reciprocal space layers, revealing the nature of the fibre-textured samples.

## Abstract 2

A simple symmetric 2D scan using a point focus beam was used to determine the fibre axis of fibre-textured satin spar samples. A geometric explanation of the ‘wing’ feature formed by the diffraction spots in 2D scans helps to visualize the nature of the reciprocal layers of the fibre-textured samples through wide-range reciprocal space mapping.

## 1. Introduction

A fibre texture forms in polycrystalline bulk samples when most crystals have one crystal orientation aligned in the same sample direction (Birkholz *et al.*, 2006 ▸). Many natural minerals exhibit an obvious fibre texture [*e.g.* asbestos, balangeroite, carlosturanite, torbernite, ulexite (Ghose *et al.*, 1978 ▸; Compagnoni *et al.*, 1983 ▸; Mellini *et al.*, 1985 ▸; Ferraris *et al.*, 1987 ▸; Ahn & Buseck, 1991 ▸)]. Satin spar is another example of naturally occurring minerals with fibre texture, famous for its ‘cat’s eye’ lustre which originates from its microstructure of well aligned fibrous gypsum crystals (Zolfaghari *et al.*, 2020 ▸). Gypsum (CaSO_4_·2H_2_O) has a monoclinic unit cell of space group *C2/c* (Comodi *et al.*, 2008 ▸). A thorough characterization of the crystallographic preferred orientation of satin spar is critical to explore its influence on dehydration behaviour (Schrank *et al.*, 2021 ▸; Wright, 2021 ▸).

Because fibre-textured (uniaxial symmetry) polycrystalline bulk materials are essentially an assembly of many small single crystals with only one of their crystallographic orientations aligned in the same direction (fibre axis), the reciprocal space of each small single crystal overlaps in such a way that they seem like one set of reciprocal space spots spun around the fibre axis. The reciprocal space circles that form are characteristic of fibre-textured (uniaxial symmetry) polycrystalline bulks. Reciprocal space circles of low Miller index have been geometrically conceived by Polanyi (1921 ▸). Polanyi linked the diffraction spot positions on a 2D detector with fibre orientations in transmission geometry, which only measures low Miller index reflections, even before the seminal introduction of the concept of reciprocal space and the Ewald sphere (Ewald, 1921 ▸).

2D X-ray diffraction (XRD) has been widely used to explore crystal orientations of non-powder samples, because it provides an additional dimension γ (also known as β) orthogonal to the 2θ direction. Pole figures measured with 2D detectors are obtained more quickly than conventional 0D/1D texture characterization, because 2D detectors measure lattice planes facing a range of zenithal angles in a single frame (Nagao & Kagam, 2011 ▸; He, 2018 ▸). The crystal orientations in fibrous samples are mostly measured in transmission geometry with an X-ray beam direction normal to the 2D detector (Polanyi, 1921 ▸), which is still applied at synchrotron beamlines and for single-crystal diffractometers collecting large 2D frames (Stribeck, 2009 ▸; Stribeck & Nöchel, 2009 ▸; He, 2018 ▸). However, natural mineral bulks are commonly measured in reflection geometry as they do not allow laboratorial X-rays to penetrate. The geometries described in this report are mostly symmetric θ/θ scans, while the transmission setup in Polanyi geometry is equivalent to a θ/−θ frame in modern laboratory diffractometers. Fibre-textured thin films were also studied in both out-of-plane and in-plane grazing-incidence diffraction geometries (Birkholz, 2007 ▸; Yokoyama & Harada, 2009 ▸; Simbrunner *et al.*, 2018 ▸). Wide-range reciprocal space mapping (WR-RSM) has been used to examine the crystal orientation between the epitaxial layer and substrates (Inaba, 2017 ▸). To determine the crystallographic orientation of the fibre axis of a bulk sample, this report proposes a single 2D scan of the reciprocal plane perpendicular to the fibre axis in reflection geometry. The reciprocal space layers of fibre-textured satin spar are visualized using the WR-RSM technique through converting the ‘wing’-shaped diffraction spots measured from 2D scans to reciprocal space.

## 2. Experiment

### 2.1. Satin spar sample

**Figure 1:**
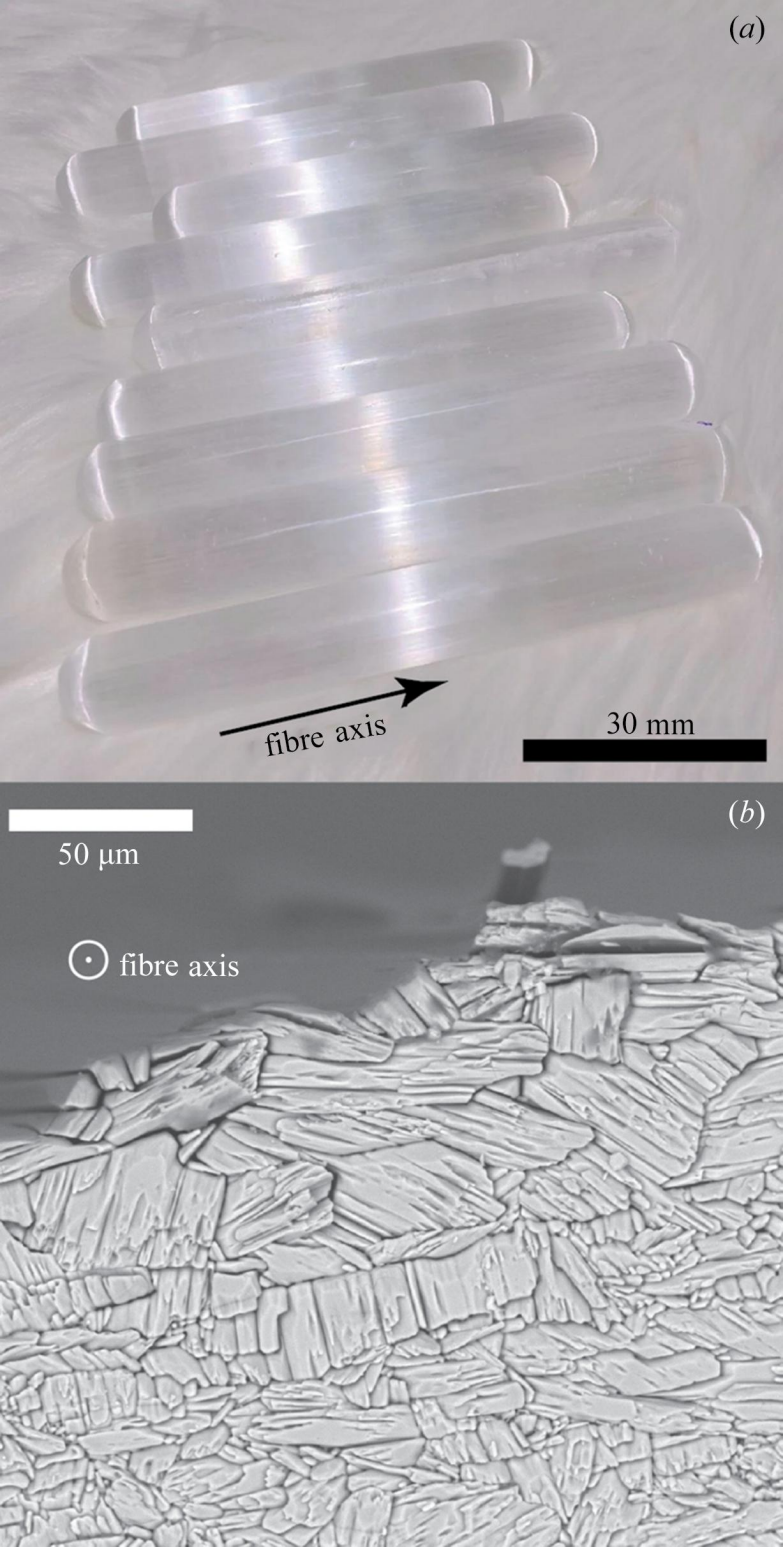
(*a*) Satin spar rods with ‘cat’s eye’ lustre perpendicular to the apparent fibre axis (marked by the arrow). (*b*) SEM image of the cutting cross-section of satin spar; ⊙ indicates that the fibre axis points towards the reader. Most fibres exhibit cross-sectional diameters between 5 and 50 µm. Note that we deliberately imaged the slightly damaged margin of the sample disc where individual fibres peel off, which makes their 3D shape visible. In our XRD analyses, the undamaged, smoothly polished disc centre was examined.

Satin spar minerals are commercially available as precious stones (Dearnaley, 2018 ▸). About 1 mm-thick discs were cut from satin spar rods perpendicular to the macroscopically visible fibre axis [the satin spar long axis, Fig. 1 ▸(*a*)]. The microstructure of the sample cross-sections is illustrated by SEM imaging in Fig. 1 ▸(*b*), demonstrating the polycrystalline nature of the material. The lattice parameters of the gypsum phase in this satin spar sample were characterized through powder diffraction (see the supporting information). No other crystalline phase was observed.

### 2.2. Symmetric θ/θ 1D and 2D scans

One thin disc sample was measured on a Rigaku SmartLab diffractometer under Cu *K*α radiation (line focus, λ = 1.54059 Å) in conventional Bragg–Brentano geometry. The scattering vector was perpendicular^1^ to the satin spar cross-section surface; no sample spinning was applied. Both the divergence slit and the detector receiving slit were fixed to 0.5°, with 5° Soller slits placed on both the primary and the secondary sides.^1^

**Figure 2:**
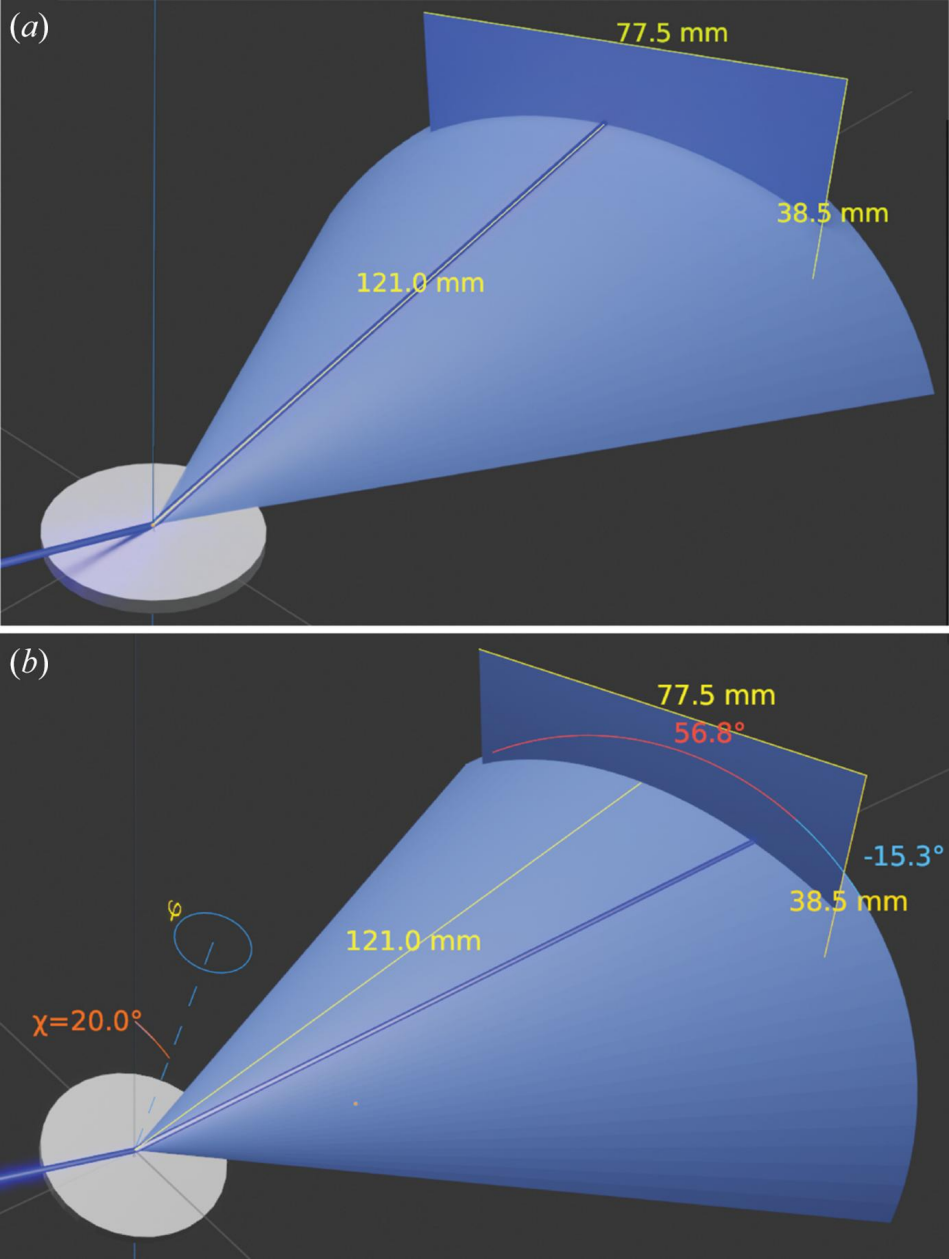
(*a*) 2D symmetric θ/θ scan with the scattering vector normal to the sample surface (satin spar cross-section). (*b*) 2D pole figure measurement (cradle χ tilt 20°, 2θ fixed to 31.1°): 2D frames for each φ rotation were recorded.

These 1D data were compared with a 2D scan in the same symmetric scan range but with a point focus beam and a 2D detector. On the same diffractometer under the same radiation wavelength, a point focus beam was created using a Rigaku CBO-f poly capillary after a CBO-PB 0.5 mm pinhole and followed by an 80 mm-long 0.8 mm-diameter collimator. The satin spar cross-section surface of the thin disc sample described in Section 2.1 was aligned using the direct X-ray beam at the centre of a χφ Eulerian cradle, which was also aligned to the centre of the goniometer. The Hypix3000 detector was used in 2D mode [(H) 38.5 mm × (W) 77.5 mm] to capture reflection intensities along the Debye cone, 121 mm away from the goniometer centre, as shown in Fig. 2 ▸(*a*). Findings of this comparison are described in Section 3.1.

### 2.3. Texture characterization: rocking scan looped on φ

Also known as ‘butterfly’ scans (Guo *et al.*, 2000 ▸), rocking scans looped on φ are commonly used for the characterization of a ‘miscut’ angle for a single-crystal surface. A miscut angle is defined as the angle between the (*hkl*) plane being measuring and the sample surface, which has been aligned to be perpendicular to the φ axis. The φ axis on the χφ Eulerian cradle refers to the sample in-plane spinning axis. Using the same instrumental setup as the 1D scan described in Section 2.2, rocking scans of a fixed 2θ = 94.77° were conducted from ω 42.4 to 52.4°, which is roughly a ±5° range for half of 2θ, at 0.1° step size, with azimuthal orientations, φ, from 0 to 355° at a 5° step size. The results of this measurement are described in Section 3.2.

### 2.4. Texture characterization: 2D pole figure

Using the same instrumental setup as described for the 2D scan in Section 2.2, the gypsum (200) (following the axis defined in ICDD PDF No. 04-015-8262) pole figure was measured by fixing 2θ_200_ = 31.1°, cradle χ tilt = 20° so that the fraction of the 200 Debye arc from zenithal angle −15.3 to 56.8° was covered at each φ rotation, as shown in Fig. 2 ▸(*b*). The measured intensities along this Debye arc in 360 φ azimuthal orientation at 1° steps were integrated using the Rigaku *2DP* software, and the resulting (200) pole figure was plotted by the Rigaku *3D Explore* software. The results of this measurement are described in Section 3.2.

### 2.5. Proposed single 2D scan to determine the fibre axis

**Figure 3:**
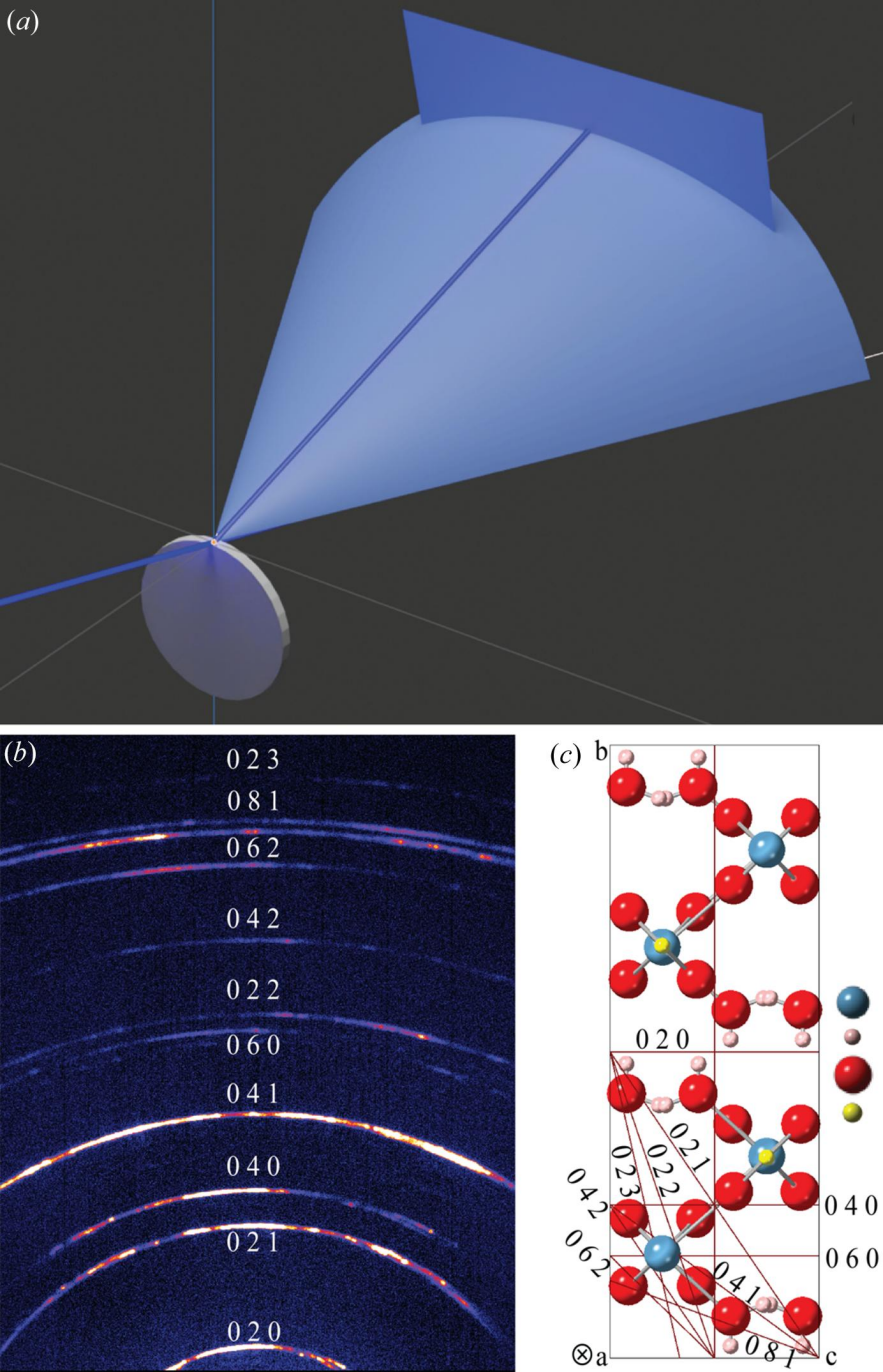
(*a*) 2D symmetric θ/θ scan with the scattering vector perpendicular to the apparent fibre axis. (*b*) 2D frame scanned from this geometry showing only 0*kl* Debye arcs. (*c*) (0*kl*) planes in the gypsum unit cell and their common parallel fibre texture axis: 〈100〉 marked with ‘⊗’. The fibre texture of crystals can be represented by spinning the unit cell around its *a* axis, where each rotation represents a crystal orientation.

A single symmetric 2D scan was collected using the same instrumental setup as the 2D scan described in Section 2.2, but aligning the apparent fibre axis of the satin spar sample along the X-ray beam direction when θ = 2θ = 0°, as shown in Fig. 3 ▸(*a*). The symmetric 2D scan in this geometry essentially collects a reciprocal plane perpendicular to the fibre axis and therefore can capture Debye arcs from most of the (*hkl*) planes parallel to the fibre axis. Indexing these planes allows us to determine the crystallographic orientation of the fibre axis, which is the crystal orientation commonly parallel to all the diffracted crystal planes. The results of this measurement are described in Section 3.3.

### 2.6. Wide-range reciprocal space mapping

Using the same instrumental setup as the 2D scan described in Section 2.2, multiple 2D frames from symmetric θ/θ scans at various cradle χ tilts were recorded. Although the χ range on a χφ Eulerian cradle normally only covers the positive direction, negative χ tilts can be achieved by rotating the sample 180° along the φ axis. The results of these measurements are described in Section 3.5.

The 2D frames collected at constant χ tilts (0 to 80° at 10° χ steps) in φ = 0°/180° and φ = 90°/270° orientations were ‘χ expanded’ in the Rigaku *2DP* software and merged into the wide-range reciprocal space map with the Rigaku *3D Explore* software. The resulting maps are discussed in Section 3.6.

## 3. Results and discussion

The flow of analyses discussed below follows a suggested logical characterization sequence for unknown bulk samples. Bragg–Brentano 1D data (Section 3.1) were collected first as the setup is readily available, followed by a symmetric 2D scan to cover the γ range out of the equatorial plane, since the prior data suffered too much from preferred-orientation effects. When a single-crystal-like diffraction frame was obtained in the 2D scan, texture analyses (Section 3.2) for the strong reflection and long reflection (at a low 2θ angle) were performed to understand the mis-cut angle as well as the texture nature. Once the fibre texture is confirmed, we propose a simple 2D scan to determine the crystallographic orientation of the fibre axis (Section 3.3). Following the derivation of the reciprocal space layers of the fibre-textured sample (Section 3.4), Section 3.5 geometrically attributes the ‘wing’ feature formed by diffraction spots in 2D scans back to the reciprocal space layers, and then Section 3.6 provides an experimental visualization using the WR-RSM technique.

### 3.1. Comparing 1D and 2D scans

**Figure 4:**
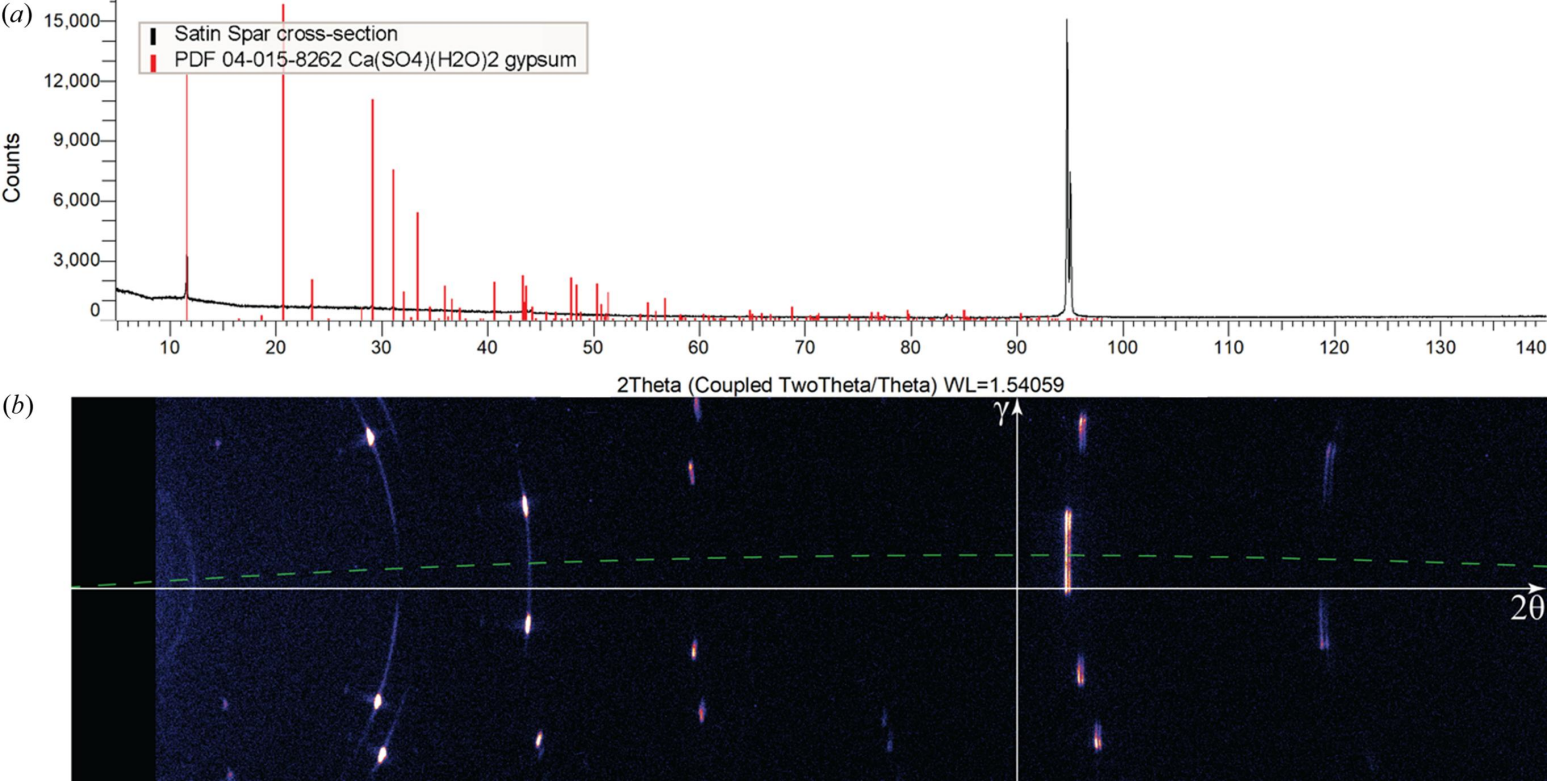
Symmetric θ/θ scans described in Section 2.2 are compared on the same 2θ axis: (*a*) 1D pattern (black data) with gypsum powder diffraction intensities in PDF 04-015-8262 (red sticks) normalized to the same maximum; (*b*) 2D frame from the same sample mounting [Fig. 2 ▸(*a*)] with a mirror 2θ line at γ = 1.7° (green dashed line).

As shown in Fig. 4 ▸(*a*), the conventional 1D curve in Bragg–Brentano geometry from the surface of the thin disc (satin spar cross-section) only features a strong peak at 94.77° 2θ. Despite the polycrystalline nature of the sample identified through SEM imaging [Fig. 1 ▸(*b*)], the strong preferred orientation of the sample does not allow any meaningful data analysis or even phase identification. Fig. 4 ▸(*b*) explains the reason for the outstanding peak at 94.77° 2θ: an elongated reflection at that 2θ angle happens to be close to the detector central line γ = 0°. The green dashed 2θ line dragged to the γ = 1.7° position in the Rigaku *2DP* software seems to be a mirror line for the reflection spots in Fig. 4 ▸(*b*).

### 3.2. Texture analysis

**Figure 5:**
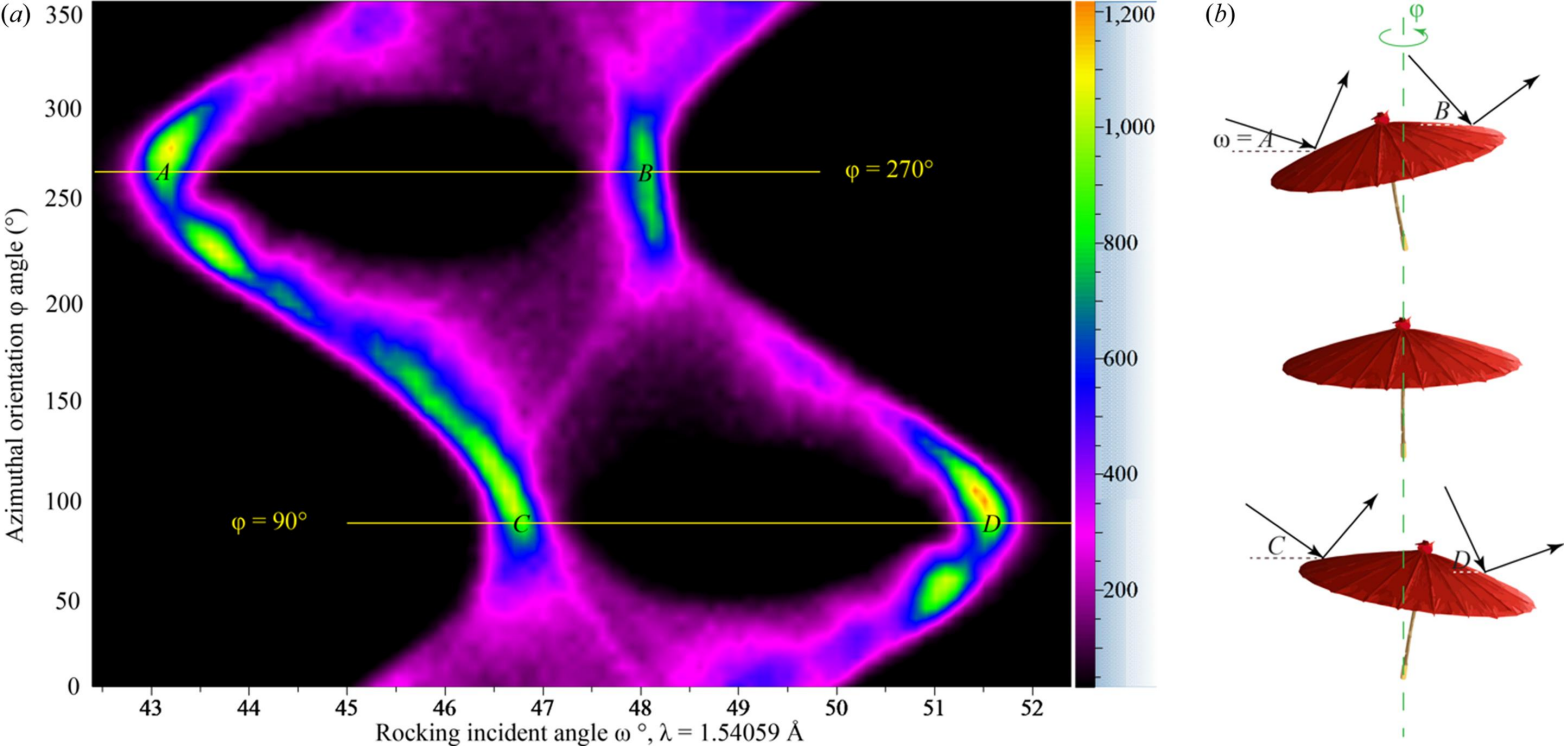
(*a*) 2D plot (φ versus ω) of all rocking curves of the reflection at 2θ = 94.77° for 0–355° azimuthal orientations at 5° φ steps. For almost every φ orientation, ‘two domains’ giving this reflection were recorded. The lowest and highest ω angles for the largest miscut domain are marked as ‘*A*’ and ‘*D*’, and those for the least miscut domain are marked as ‘*C*’ and ‘*B*’. (*b*) Analogous representation of the fibre-textured polycrystalline sample in this ‘miscut’ measurement using a Chinese oil paper umbrella.

To find the crystal plane orientation for the elongated reflection at 2θ*_hkl_* = 94.77° [Fig. 4 ▸(*b*)], the ‘miscut’ measurement described in Section 2.3 was conducted. The resulting rocking scans at different φ orientations are plotted in 2D mode [Fig. 5 ▸(*a*)]. Instead of stacking all rocking curves together as a ‘butterfly diagram’, which loses azimuthal resolution, Fig. 5 ▸(*a*) disperses them according to their φ angles. It is obvious that for every φ angle ‘two domains’ containing this *hkl* reflection were observed, indicating that the sample should obey an axial rotational texture symmetry rather than any mirror texture symmetry. Among these domains, the largest miscut angle can be calculated as |*D* − *A*|/2 ≃ 4.2°, while the smallest miscut angle is |*C* − *B*|/2 ≃ 0.7°. All the other domains should have miscut angles in between these bounds. The angle between the fibre axis and the φ axis can be calculated as (*C* − *A*)/2 = (*D* − *B*)/2 ≃ 1.75°. An analogous visualization of the arrangement of (*hkl*) planes in fibre texture can be obtained using a representation of an ancient Chinese oil paper umbrella, as shown in Fig. 5 ▸(*b*). The incident angles ‘*A*’ and ‘*B*’ were achieved when the ‘umbrella’ was rotated to the left (anti-clockwise), while ‘*C*’ and ‘*D*’ were achieved when it was rotated to the right.

**Figure 6:**
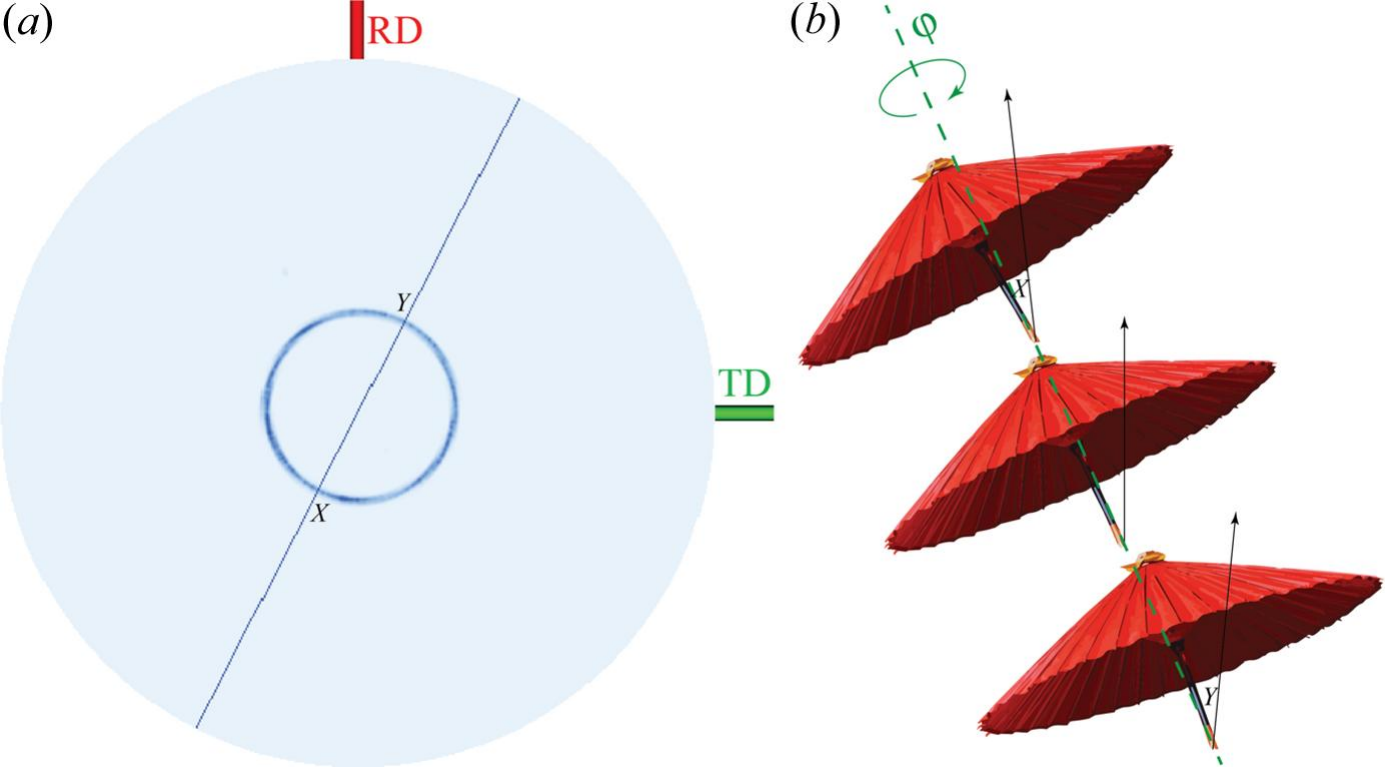
(*a*) Gypsum (200) pole figure of the satin spar sample showing fibre texture. The maximum and minimum zenithal angles are marked as ‘*X* ’ and ‘*Y* ’, respectively. (*b*) Analogous representations of the gypsum (200) plane in fibre texture illustrating the formation of the maximum and minimum zenithal angles.

To better understand the crystal orientation, a pole figure measured for the gypsum 200 reflection (2θ_200_ = 31.1°) as described in Section 2.4 is shown in Fig. 6 ▸(*a*). The 200 reflection was chosen because of the strong diffraction of the lowest 2θ angle observed in Fig. 4 ▸(*b*), which warrants the widest zenithal angle coverage in the 2D pole figure measurement. Fig. 6 ▸(*a*) unambiguously verifies the fibre-textured nature of the cross-sectional satin spar disc. The angle between the fibre axis and (200) plane can be calculated as |*X* + *Y*|/2 ≃ 24°. The tilt angle between the fibre axis and the φ axis can be calculated as |*X* − *Y*|/2 ≃ 1.7°, which aligns well with that derived from the ‘miscut’ scans from the reflection at 2θ*_hkl_* = 94.77°.

### 3.3. Fibre axis definition

To find the crystal orientation of the fibre axis, the 2D frame collected from the edge of the satin spar thin disc described in Section 2.5 is shown in Fig. 3 ▸(*b*). Indexing of the measured Debye arcs suggests that only 0*kl* reflections were present. Therefore, the fibre axis is determined to be the crystal orientation commonly parallel to all of these planes, *i.e.* the 〈100〉 zone axis or *a* axis, as illustrated in Fig. 3 ▸(*c*). The relatively continuous intensities along these Debye arcs suggest that the gypsum crystal domains are relatively randomly distributed around the fibre axis. In the gypsum unit cell, the angle between 〈100〉 or the *a* axis and the (200) plane is 24.1°, which aligns well with the findings from the 2D pole figure measurement analysed in Section 3.2.

Note that this method cannot be achieved in the Polanyi (1921 ▸) transmission geometry, because fibre samples cannot be tilted to 90° in that setup, or a very thin cross-section perpendicular to fibre axis needs to be prepared to allow the X-ray beam to penetrate. Modern laboratory diffractometers have both X-ray tubes and detectors that can be rotated away from the horizontal direction, enabling the measurement of the reciprocal plane perpendicular to the fibre axis. The current geometry can be easily applied to the surface of other mineral rods, to find the crystallographic orientation of their apparent fibre axis, without any cutting.

### 3.4. Reciprocal space of fibre-textured satin spar

**Figure 7:**
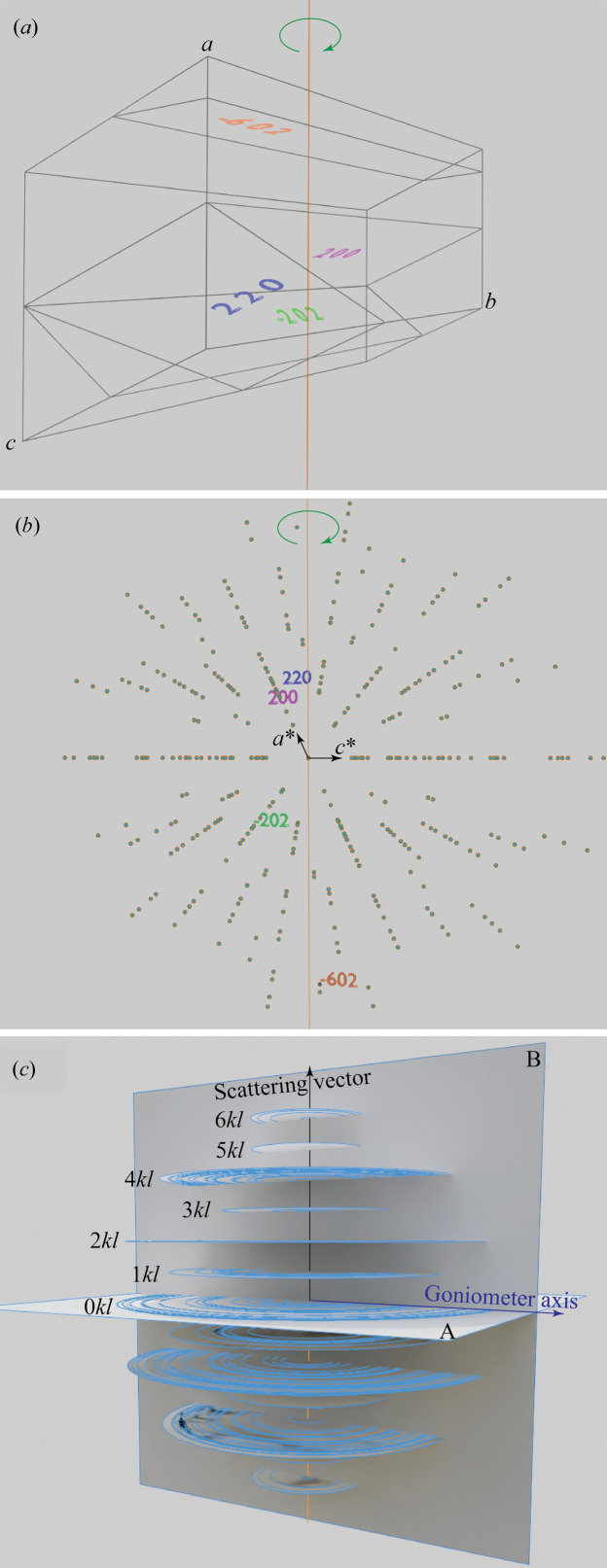
(*a*) Gypsum unit cell with four selected crystal planes labelled; the fibre axis is parallel to the *a* axis direction. (*b*) Gypsum reciprocal space with labelled reciprocal spots corresponding to the planes in (*a*). (*c*) All reciprocal space spots in (*b*) spun around the fibre axis, then cut by the scattering vector plane (*B*, also parallel to the goniometer axis) of a symmetric 2D scan. All the measured reciprocal spots form parallel intersection lines which are perpendicular to the fibre axis. Measuring the reciprocal plane *A* perpendicular to the fibre axis [Fig. 3 ▸(*b*)] allows us to determine the crystallographic orientation of the fibre axis.

The gypsum unit cell and its corresponding reciprocal space are illustrated in Figs. 7 ▸(*a*) and 7 ▸(*b*), with four selected planes and corresponding reciprocal spots labelled. Crystal domains in fibre texture along the *a* axis can be represented by spinning the unit cell around the *a* axis; each rotation represents a domain orientation. Similarly, the corresponding reciprocal space should also be spun around the fibre axis, which results in concentric circles, as shown in Fig. 7 ▸(*c*). The upper half of Fig. 7 ▸(*c*) is in mirror symmetry to its lower half. Any pair of upper and lower reciprocal circles form the ‘Polanyi sphere’ (Polanyi, 1921 ▸; Stribeck, 2009 ▸). The measurement in Fig. 3 ▸(*b*) collected part of the reciprocal plane *A* in Fig. 7 ▸(*c*), which allows us to determine the crystallographic orientation of the fibre axis.

However, if the fibre axis of the sample is aligned in the direction of the scattering vector in symmetric 2D scan mode, only reciprocal spots that lie on the scattering vector plane [plane *B* in Fig. 7 ▸(*c*)] can be measured. Therefore, all the measured reciprocal spots should lie on parallel intersection lines, which are perpendicular to the fibre axis. The ( 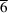 02) or (60 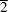 ) crystal planes are almost perpendicular to the fibre axis, *i.e.* the *a* axis, as shown in Fig. 7 ▸(*a*). Therefore the 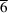 02 or 60 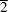 reciprocal spots are closest to the fibre axis in Fig. 7 ▸(*b*), and form the circle with the smallest radius in Fig. 7 ▸(*c*). This explains why an elongated diffraction spot is observed at 2θ_ 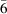 02_ = 94.77° in Fig. 4 ▸(*b*).

### 3.5. The ‘wing’ features of diffraction spots

**Figure 8:**
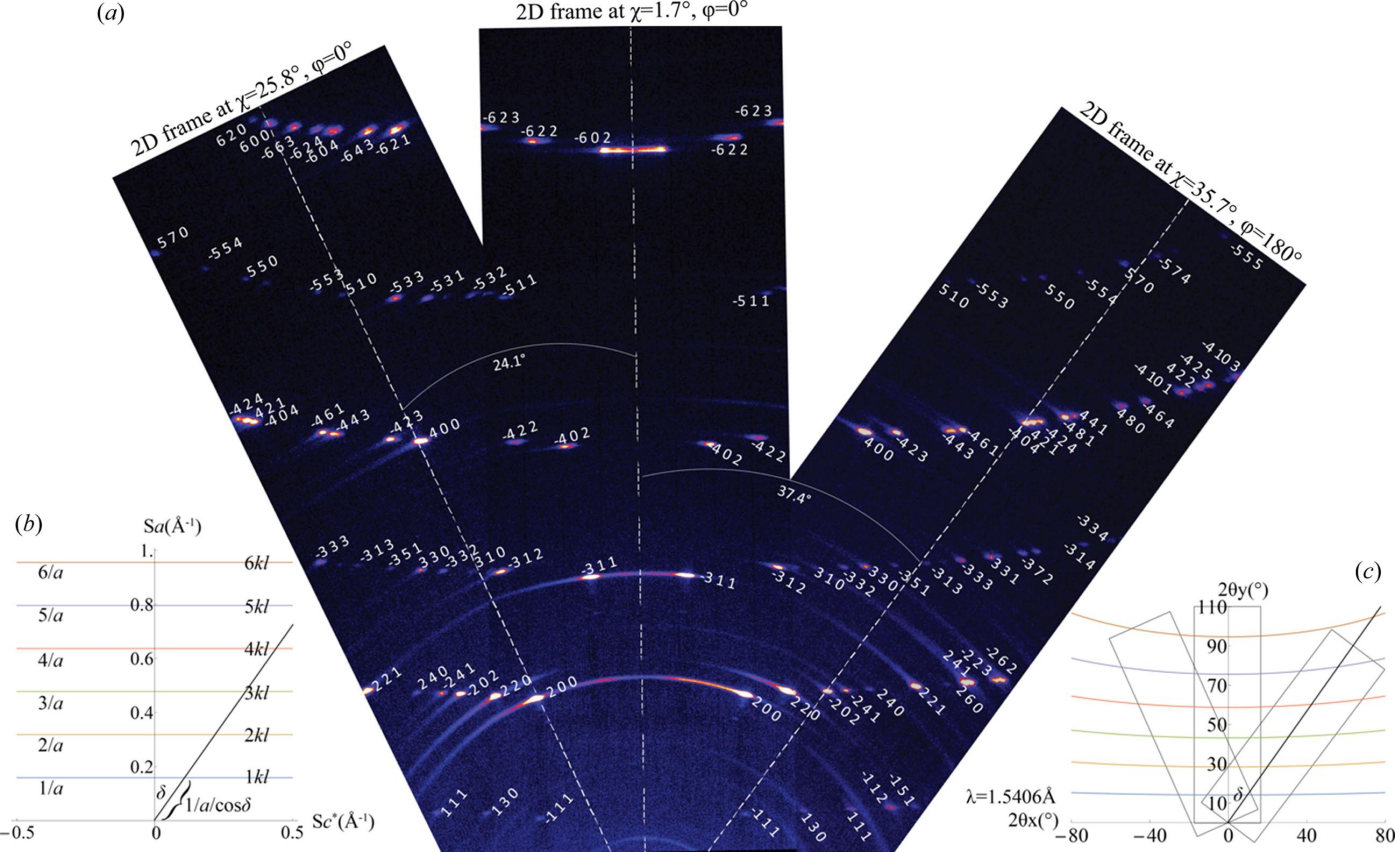
(*a*) 2D frames spliced according to their χ tilt angles. All the diffraction spots are indexed and form *nkl* (*n* = 1, 2, 3, 4, 5, 6,…) ‘wings’. (*b*) Reproduction of the reciprocal space in Fig. 7 ▸(*c*) viewed from the b* direction. The distance between the spots on the *nkl* equidistant parallel lines and the origin can be calculated from the line spacing ‘*n*/*a*’ and the spot’s zenithal angle ‘δ’ from the fibre axis. (*c*) The equidistant parallel lines in (*b*) converted to 2θ space on 2D detectors, under Cu *K*α radiation. The scan ranges of the three 2D frames are outlined according to their corresponding χ tilt angles in (*a*).

The 2D frames collected in θ/θ scans from the cross-section surface of the satin spar thin disc at both positive and negative χ tilts on the χφ Eulerian cradle, as described in Section 2.6, are shown in Fig. 8 ▸(*a*). Interestingly, the diffraction spots on the 2D detector form a ‘Seraph wings’ pattern. On the basis of the reciprocal space concentric circles of fibre-textured satin spar derived from the analysis in Section 3.4 and Fig. 7 ▸(*c*), the parallel intersection lines representing *nkl* (*n* = 1, 2, 3, 4, 5, 6) layers are drawn in Fig. 8 ▸(*b*). Converting the reciprocal space dimension *s* (Å^−1^) on these six lines into the 2θ space on the 2D detector shown in Fig. 8 ▸(*c*) requires the following equations:

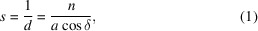




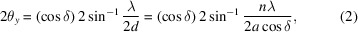




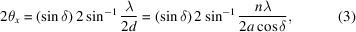

where *d* represents the *d* spacing (Å), *a* is the gypsum unit cell *a* axis length (Å), δ is the angle (°) of each reciprocal space spot from the fibre axis, λ is the radiation wavelength, and 2θ_*x*_ and 2θ*_y_* are the two orthometric dimensions on the 2D detector. Using the above equations, the simulated lines on which the diffractions spots lie and the 2D detector positions in Fig. 8 ▸(*c*), we successfully estimated the real diffraction spots collected in Fig. 8 ▸(*a*).

### 3.6. WR-RSM of fibre-textured satin spar

**Figure 9:**
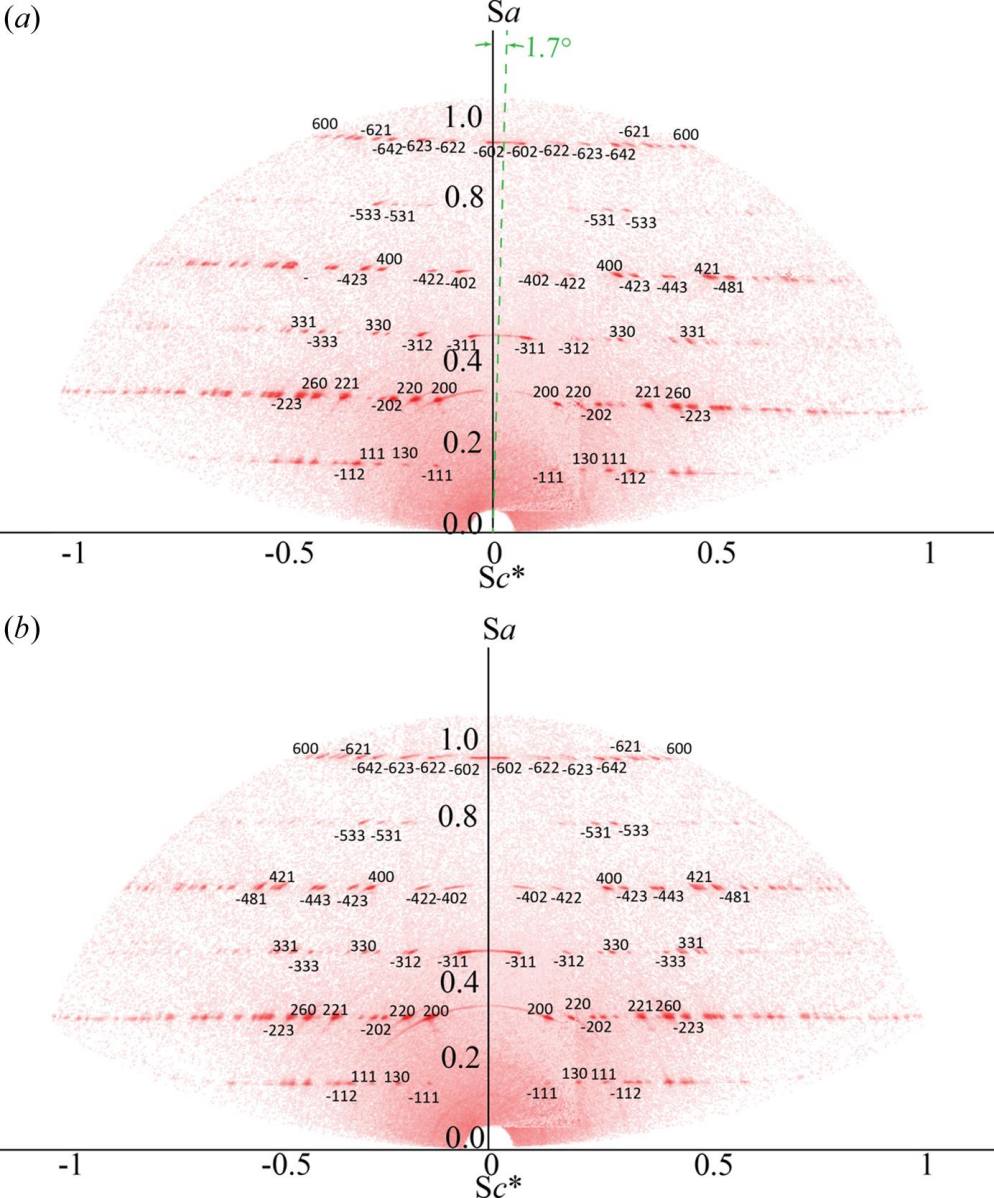
Full reciprocal space of the fibre-textured satin spar sample at (*a*) φ = 0° and (*b*) φ = 90°.

The upper half of the reciprocal plane [Fig. 7 ▸(*c*), plane *B*] of the fibre-textured satin spar mapped using the WR-RSM technique, as described in Section 2.6, is shown in Fig. 9 ▸. These plots verified the estimated intersecting spots of the reciprocal spacing concentric circles and the scattering vector plane [Fig. 7 ▸(*c*), plane *B*]. As characterized in Section 3.2, the fibre axis (*i.e.* the *a* axis) has an ∼1.7° tilt from the φ axis, which is seen in the φ = 0° orientation of the reciprocal space map in Fig. 9 ▸(*a*).

## 4. Conclusions

The application of 2D XRD (point focus X-ray beam and 2D detector) on fibre-textured natural satin spar (gypsum phase) has been demonstrated in this paper. A single 2D scan collected from the sample with its apparent fibre axis placed along the X-ray beam direction at θ = 2θ = 0° provides sufficient information to determine the crystallographic orientation of the fibre axis. This approach is readily applicable to other natural mineral rods with minimum sample preparation. The ‘wing’ feature formed by diffraction spots observed on a 2D detector has been explained geometrically. The technique of WR-RSM was applied to convert the ‘wing’ featured diffraction spots into reciprocal space layers, revealing the nature of the fibre-textured samples.

## Supplementary Material

Sample characterization by powder diffraction. DOI: 10.1107/S1600576724000529/xx5039sup1.pdf

## Footnotes

1 Strictly, the range of scattering vector directions covered by 1D and 2D detectors is approximately ±(PSD opening angle)/4 around the scattering vector of their central pixel. In the case where a narrower range of directions is required, one can increase the sample-to-detector distance (StDD), or scan using pixels in fewer central channels.
