# Supplementary material for: Visualizing the fibre texture of satin spar using laboratory 2D X-ray diffraction
Source: J Appl Crystallogr. 2024 Feb 12;57(Pt 2):240–7. doi: 10.1107/S1600576724000529 (PMC11001398; doi:10.1107/S1600576724000529)
Supplement: Supplementary file 1 [file j-57-00240-sup1.pdf]

JOURNAL OF
APPLIED
CRYSTALLOGRAPHY

Volume 57 (2024)

Supporting information for article:

Visualizing the fibre texture of satin spar using laboratory 2D X-ray diffraction

Xiaodong (Tony) Wang, Christoph Schrank and Michael Jones

S1. Powder Diffraction

To precisely measure the lattice parameters of the gypsum phase in above satin spar samples, a fraction of the satin spar was grinded into powder and spiked with NIST SRM 676a corundum powder as the internal standard. The powder diffraction pattern of the mixture measured in conventional Bragg-Brentano geometry under CoKα radiation (λ = 1.7889 Å) were matched against ICDD PDF-5+ 2024 database using DIFFRAC.EVA v7, which best matches with PDF# 04-015-8262 (Comodi *et al.*, 2008). Pawley refinement of gypsum unit cell and sample displacement error were conducted in DIFFRAC.TOPAS v7 against the fixed lattice parameters of SRM 676a from its certificate (Figure S1). The refined lattice parameter of this Satin Spar powder is shown in Table S1.

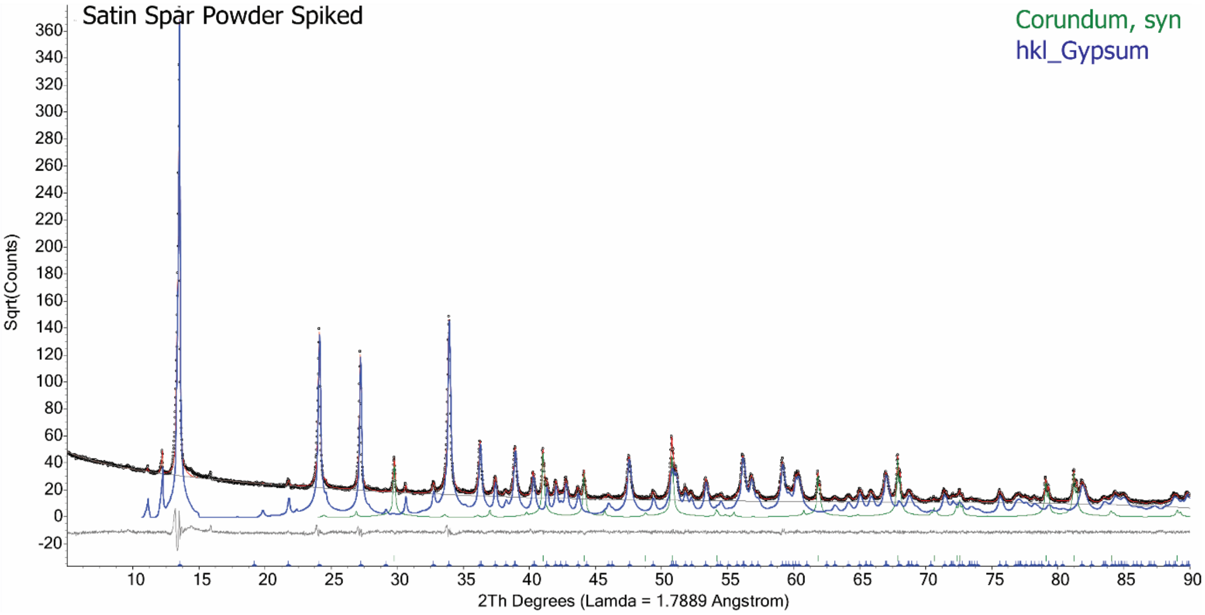

Pawley refinement of Gypsum unit cell with sample displacement, peak profile, and background refined; corundum lattice parameters fixed to the NIST SRM 676a certificate. Rwp = 10.23%; GOF = 2.99.

Table S1 Summary of the refined unit cell of gypsum phase in the satin spar sample. The number in the bracket is calculation error for the last decimal place.

Space group	*a* (Å)	*b* ()	*c* (Å)	*β* (°)
C2/c	6.2852(3)	15.2056(4)	5.6784(3)	114.100(4)
